# Comparison of Dental Findings with Computed Tomographic and Clinical Examination in Patients with End-Stage Heart Failure

**DOI:** 10.3390/jcm13185406

**Published:** 2024-09-12

**Authors:** Cordula Leonie Merle, Julia Gocke, Patrick Seitz, Matthias Gutberlet, Diyar Saeed, Rainer Haak, Dirk Ziebolz, Robin Fabian Gohmann, Gerhard Schmalz

**Affiliations:** 1 Department of Prosthetic Dentistry, UKR University Hospital Regensburg, 93042 Regensburg, Germany; 2 Department of Cariology, Endodontology and Periodontology, University of Leipzig, 04103 Leipzig, Germany; 3 Heart Center Leipzig, University Department for Cardiac Surgery, 04289 Leipzig, Germany; 4 Department of Diagnostic and Interventional Radiology, Heart Center Leipzig, 04289 Leipzig, Germany; 5 Medical Faculty, University of Leipzig, 04103 Leipzig, Germany

**Keywords:** heart disease, heart transplantation, oral health, radiographs, computed tomography

## Abstract

**Background**: This study aimed to evaluate the diagnostic value of pre-existing computed tomography (CT) examinations for the detection of dental pathologies compared with clinical dental examination in patients with end-stage heart failure. **Methods**: For this purpose, 59 patients with end-stage heart failure and pre-existing non-dental CT images of the craniofacial region were included. Virtual orthopantomograms (vOPG) were reconstructed. Dental pathologies were analyzed in vOPG and source-CT images. Imaging and clinical findings less than 6 months apart were compared (*n* = 24). **Results**: The subjective image quality of vOPG was more often rated as insufficient than CT (66%; 20%; *p* < 0.01). Depending on examination (CT, vOPG or clinic), between 33% and 92% of the patients could require dental intervention such as treatment of caries and periodontitis or tooth extraction. vOPG led to a higher (80%) prevalence of teeth requiring treatment than CT (39%; *p* < 0.01). The prevalence of teeth requiring treatment was similar in CT (29%) and clinic (29%; *p* = 1.00) but higher in vOPG (63%; *p* < 0.01). CT (stage 3 or 4: 42%) and vOPG (38%) underestimated the stage of periodontitis (clinic: 75%; *p* < 0.01). **Conclusions**: In conclusion, available CT images including the craniofacial region from patients with end-stage heart failure may contain valuable information regarding oral health status. The assessability of vOPGs might be insufficient and must be interpreted with caution.

## 1. Introduction

Heart failure (HF) is a common cause of morbidity and mortality [1]. HF is considered to be the second leading cause of death among women and the fourth among men [2] with a prevalence of about 4% and a mortality rate of 16.3% within the first two years after diagnosis [3]. During the course of disease, circulatory support with a left ventricular assist device (LVAD) may frequently be needed, either as a bridge to heart transplantation (HTx) or destination therapy [4]. Patients with HTx and LVAD are at increased risk for infectious complications due to immunosuppression [5], driveline-infections [6], or co-morbidities [7].

Avoiding infectious complications in HF patients is crucial. Insufficient oral health has been described as a potential risk factor for infectious complications [8], hospitalization, and overall mortality [9]. Consequently, dental clearance before HTx is recommended [10]. Nevertheless, several studies have shown poor oral health status in individuals with severe heart diseases [11,12,13,14,15], even in patients with a high risk for infections due to LVAD or HTx [12,13,14]. More than 80% had overall treatment need and especially periodontal treatment need was common [12,14]. While there is a lack of adequate dental care, these patients undergo comprehensive cardiological diagnostics, often including computed tomography (CT) [16]. Carotid angiography and screening for infectious foci frequently require scans of the head and neck region in patients with HF, LVAD, and HTx [17,18]. Consequently, the first information about the oral health status of these patients may already be available in these cross-sectional images.

A first proof of concept showed the utility of dental panoramic 2D reconstructions made out of non-dental CT for the detection of dental pathologies [19]: reconstructed virtual orthopantomogram (vOPG) were interpreted significantly faster and with a higher accuracy than CT but only in CT were all periapical lesions detected [19]. On the other hand, it has been shown that dental pathologies may not always be diagnosed in CT imaging, even if already causing maxillary sinusitis [20]. Until now, there has been no systematic evaluation of the potential of those CT images to detect oral foci in HF patients. Moreover, it is unknown whether or not CT images and reconstructed virtual orthopantomogram (vOPG) provide additional information compared with clinical dental examination alone.

This retrospective study aimed to evaluate the diagnostic value of pre-existing CT. The CT scans and vOPG were evaluated and the amount of rated oral health findings were compared with documented clinical examinations. A subjective rating of the assessability of CT and vOPG and their association to CT parameters was analyzed.

## 2. Materials and Methods

This study is a retrospective analysis of patients’ data available from a previous prospective study conducted by the Department of Cariology, Endodontology and Periodontology, University of Leipzig and the Leipzig Heart Centre [12,14]. The study was conducted according to the guidelines of the Declaration of Helsinki and was approved by the Ethics Committee of the Medical Faculty of the University of Leipzig (protocol code 414/16-ek, date of approval: 9 February 2017). This approval was initially granted for a prospective study and also covers this subsequent retrospective analysis. Informed consent was obtained from all subjects as part of the initial prospective study.

### 2.1. Patients

**Figure 1:**
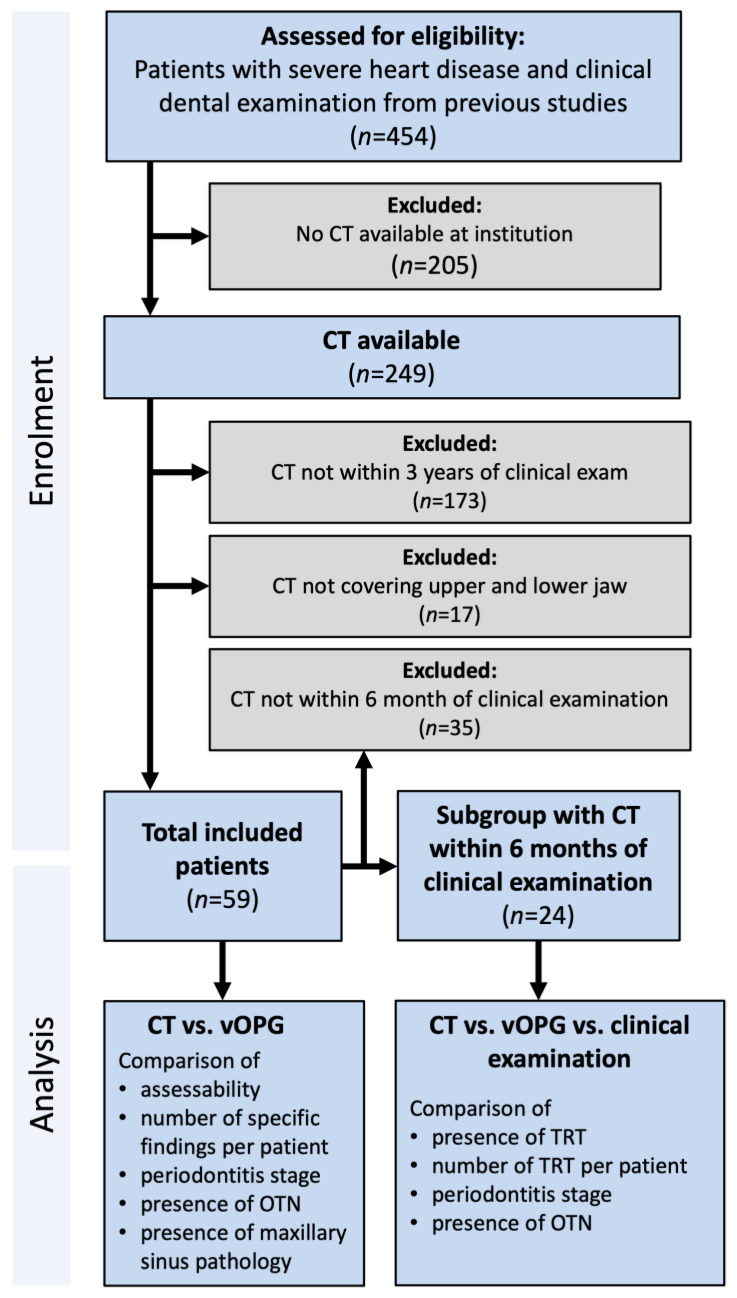
Flowchart of the study population explaining patient selection according to availability of image records; CT: computed tomography examination; OTN: overall treatment need; TRT: teeth requiring treatment; vOPG: virtual orthopantomogram.

For this study, the medical records of 454 patients with severe heart disease that had received a clinical dental examination as part of a previous study at the Department of Cardiothoracic Surgery, Leipzig Heart Centre [12,14] were screened. All patients had either received an HTx, LVAD or had end-stage heart failure and were candidates for LVAD therapy and/or HTx. All patients had received a clinical dental examination between May 2017 and August 2018. Medical records were screened for the presence of a CT at Leipzig Heart Centre (Figure 1). 

The inclusion criteria were the availability of a CT in the database of the Heart Centre Leipzig independent of the clinical indication containing the upper and lower jaw in its scan range and being within three years of the clinical dental examination. No further exclusion criteria were applied. 

For 249 patients of the cohort, CT images were available in the database of the Heart Center. However, 173 patients were excluded because the available CT images were not within three years of the clinical dental examinations. A further 17 patients were excluded because the CT images did not cover the upper and lower jaw (Figure 1).

### 2.2. Image Acquisition, Reconstruction, and Radiographic Assessment

#### 2.2.1. CT

All CT scans were performed using a second generation dual-source scanner (SOMATOM Definition Flash, Siemens Healthineers, Erlangen, Germany). The scan and reconstruction parameters of each CT (tube voltage [kV], slice thickness [mm], tube current-time product [mAs] and kernel) were recorded. The CT examinations were analyzed in the picture archiving and communication system (PACS; Sectra IDS7, version 23.2.0.5047, Sectra AB, Linköping, Sweden). All functions of the PACS, including multiplanar reformations and windowing, were available during analyses.

#### 2.2.2. Virtual Orthopantomogram (vOPG)

**Figure 2:**
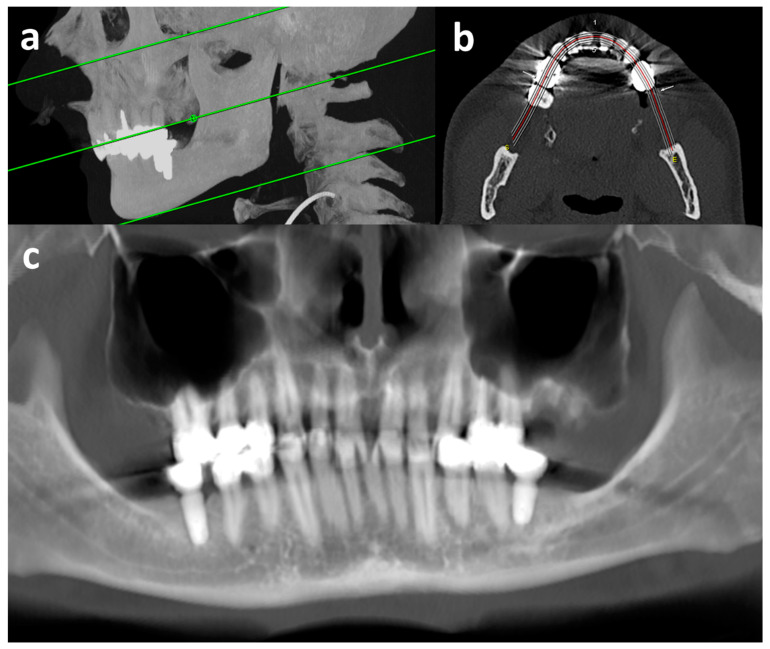
Reconstruction of vOPG from CT. Sagittal maximum intensity projection (**a**) for level selection of upper and lower limits and occlusal plain (green lines), para-axial plain (**b**) for contour selection of alveolar processes (red line: center of reconstruction), and margin selection of maximum intensity projection for the vOPG (outer white lines), and final vOPG (**c**) with very good image quality; CT = computed tomography examination; vOPG = virtual orthopantomogram.

**Figure 3:**
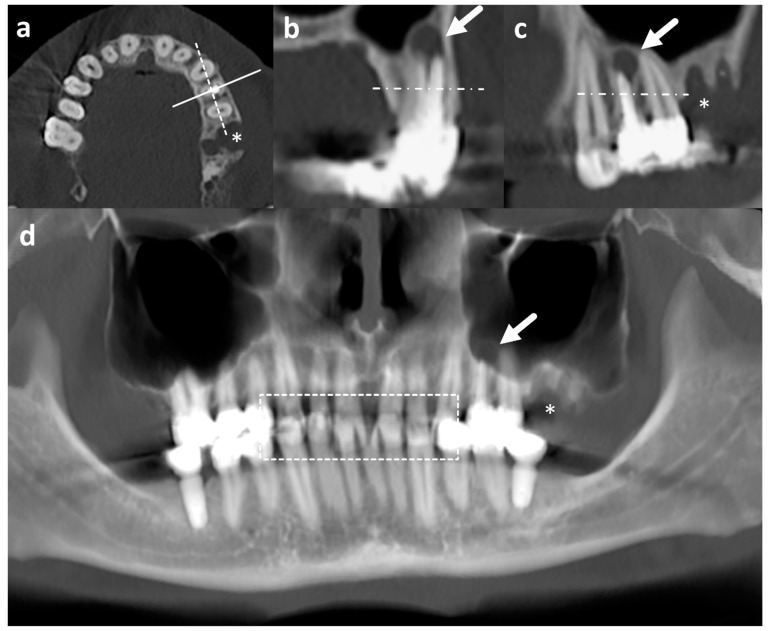
Dental part of CT images in axial (**a**), sagittal (**b**), and coronal plane (**c**), and vOPG (**d**) of the same patient. The solid and dashed line in a corresponds to the plane of sagittal (**b**) and coronal (**c**) reconstructions reconstructed in 1 mm slice thickness, respectively. Note the periapical abscess at the left maxillary first premolar with root canal treatment (arrow in (**b**–**d**)) is much more conspicuous on CT than vOPG. The asterisk (*) marks a defect after tooth extraction (**a**,**c**,**d**). The dashed box in d contains areas of overlap due to closed jaws during examination impairing evaluability particularly of vOPG. Horizontal streak artefacts may be noted in all reconstructions parallel to the image acquisition plane in all reconstruction resulting from metal implants. CT = computed tomography examination; vOPG = virtual orthopantomogram.

vOPGs were reconstructed (Figure 2 and Figure 3d) from the thinnest axial reconstructions available with a dedicated post-processing software (syngo.via, version VB60A HF03, Siemens Healthineers, Erlangen, Germany; workflow: dental). The creation of a vOPG lasted five to ten minutes. For this, a dentist manually set a plane parallel to the occlusal plane and drew a line along the occlusal surfaces of the maxillary and mandibular row of teeth. In order to create the vOPG, a maximum intensity projection at a six millimeter slice thickness was reconstructed perpendicular to this line (Figure 2). All vOPGs were reconstructed by the same dentist after prior training with an experienced radiologist.

#### 2.2.3. Radiological Evaluation

All CT and vOPG exams were evaluated by one calibrated dentist on calibrated diagnostic monitors between November 2020 and February 2021. For evaluation of CT, all planes (axial, sagittal, and coronal, Figure 3a–c) were considered. The evaluation of the images lasted on average 20 min (CT) and ten minutes (vOPG), respectively. 

The calibration process of the dentist was conducted with another experienced dentist and consisted of a collaborated evaluation of ten images and two rounds of evaluating five images at a time separately with subsequent discussion. Temporal distance between the evaluations of the different examinations (CT and vOPG) was at least one week. Before the calibration, both dentists underwent training at the radiological department over several weeks to familiarize themselves with CT-image reconstruction and analysis. A high interrater agreement was reached after the calibration process (kappa > 0.9).

#### 2.2.4. Assessability

Assessability of CT and vOPG was rated by the perception of the evaluating dentist on a 3-point scale (assessable, partially assessable, or not assessable). Assessability was evaluated separately for the following aspects: caries, sufficiency of restorations, remaining roots, apical lesions, presence and quality of root canal treatment, impacted teeth, periodontal conditions (horizontal and vertical bone loss), and presence of pathologies in the maxillary sinuses. For each aspect, only those CTs and vOPGs were rated that displayed the corresponding structures: at least one tooth for caries, apical lesions, and presence of root canal treatment, at least one erupted tooth for horizontal and vertical bone loss, at least one restoration for sufficiency of restorations, or at least one tooth with root canal treatment for quality of root canal treatment. All CTs and vOPGs were rated for remaining roots, impacted teeth, and maxillary sinus pathologies as the area of the bone and the maxillary sinus was included in due to the inclusion criteria (upper and lower jaw in the scan range). If the respective parameters were rated as partially assessable or not assessable, the supposed predominating cause was recorded as one of the following: resolution, metal artefacts, missing areas (due to the field of view during reconstruction of the CT), wrong level selection (due to wrong line selection during reformation of the vOPG), or overlapping areas (due to closed mouth during CT). 

The association of assessability and CT reconstruction parameters (slice thickness [≤0.75 mm] and kernel hardness [≥45]) were tested in both CT and vOPG.

### 2.3. Dental and Periodontal Treatment Need

#### 2.3.1. CT and vOPG

Required treatment was evaluated separately for CT and vOPG for the following categories:

- tooth with need for restoration due to caries or insufficient restorations;
- severely damaged tooth (tooth with more than 50% damage of the crown);
- remaining root(s) (tooth without crown);
- apical lesion at tooth with/without root canal treatment;
- tooth partial impacted by bone or soft tissue.

The presence of at least one of these findings was rated as overall treatment need (OTN). The sum of these findings was counted as the number of OTN. The presence of teeth with need for restorations, severely damaged teeth, and remaining roots were summarized as teeth requiring treatment (TRT).

Completely bony impacted teeth, periodontitis stage according to maximal horizontal bone loss (stage 1: <15%, stage 2: 15–33%, stage 3 or 4: >33%), presence of vertical bone loss (≥3 mm) [21], and presence of any pathology in the maxillary sinuses were also assessed. 

#### 2.3.2. Clinical Examination

The clinical examinations had taken place between May 2017 and August 2018 at Leipzig Heart Centre as part of a previous study. Setting and methods of the clinical examination are described in detail in the corresponding manuscript by Binner et al. 2019 [12]. The following parameters were extracted from the corresponding data set:

- number of teeth with carious cavitation (D-T) according to WHO [22] (including all stages with treatment need as decayed teeth, insufficient restorations, severely damaged teeth, and remaining roots);
- periodontal inflamed surface area (PISA) [23];
- periodontitis diagnosis based on detailed periodontal chart (periodontal probing depth, attachment loss, furcation involvement, tooth mobility) with staging and grading [24];
- presence of periodontal probing depth of over 3 mm [21] indicating periodontal treatment need;

Number of D-T, respectively; the presence of D-T > 0 was classified as clinical TRT. OTN was stated if patients showed TRT and/or periodontal treatment need.

### 2.4. Comparisons

Assessability of CT and vOPG were compared for all patients if the image displayed the corresponding structures. For treatment need, the number of the different aspects and number and presence of OTN were compared between CT and vOPG for all patients. If CT and clinical exam were less than 6 months apart, CT and vOPG were compared with the clinical dental examination. Here, number and presence of TRT and presence of OTN (CT/vOPG: presence of TRT or apical lesions or partially impacted teeth; clinic: presence of TRT or periodontal treatment need) were compared. 

### 2.5. Statistical Analysis

Metric variables are reported as mean ± SD when normally distributed or as median (inter quartile range [IQR]) when data were not normally distributed; categorical variables are reported as count (percentage). Kolmogorov–Smirnov test was used to determine (non-)normal distribution for metric variables. For group comparisons of non-normally distributed metric variables, a Wilcoxon test was used, or for more than two groups, Friedman test was used. Ordinal variables were compared by sign test, dichotomous variables by McNemar’s test, or by sign test for more than two groups. 

Pearson’s chi-squared test was used for the analysis of possible associations between the assessability and the presence of CT reconstruction parameters (slice thickness and kernel hardness). 

Statistical calculations were performed with the software package SPSS (SPSS for Windows, version 24.0, SPSS Inc., Chicago, IL, USA). The level for significance was defined at *p* < 0.05.

## 3. Results

### 3.1. Patients

All CT scans including the head and neck region of the study group were screened for suitability and a total of 59 patients were included (Figure 1). The mean age of the 59 included patients was 54.9 ± 10.0 years and 91.5% were male. vOPG could be constructed from CT for all patients. For 24 patients, clinical data were available within 6 months of CT, allowing for a comparison of findings between CT, vOPG, and clinical examination. Patient characteristics between patients with a clinical examination < 6 months and ≥6 months of CT did not differ significantly (*p* ≥ 0.09). Detailed patient characteristics are presented in Table 1. 

### 3.2. Assessability

Assessability by aspects is presented in Table 2. CT was more frequently rated as assessable or partially assessable compared with vOPG for the evaluation of caries, apical lesions, the presence of root canal treatment, impacted teeth, maximal horizontal periodontal bone loss, periodontal vertical bone loss, and maxillary sinus pathology (*p* < 0.01).

The most frequent cause of impaired assessability were metal artefacts (CT: 84.8%; vOPG: 83.1%), insufficient resolution (CT: 20.3%; vOPG: 66.1%), missing areas (CT: 8.5%; vOPG: 27.1%), overlapping areas (CT: 35.6%), and wrong level selection (vOPG: 23.7%).

For most aspects of assessability, a thinner slice thickness, a harder kernel, or a combination of the two were associated with a better rating (Table 3).

### 3.3. Dental Findings

The interrater agreement and intrapersonal reproducibility of the evaluation of CT and vOPG was high (kappa > 0.9), respectively.

Dental findings according to CT and vOPG are reported in detail for all aspects in Table 4. On CT, each patient had an average of 1.6 OTN and 0.7 apical lesions; 39.0% of exams had OTN and 44.1% had severe periodontitis (stage 3 or 4). Compared with CT, vOPG had significantly more findings of caries or insufficient restorations (*p* < 0.01), severely damaged teeth (*p* = 0.04), and findings requiring treatment (*p* < 0.01). Findings in the maxillary sinuses were more frequently observed on CT (CT: 27 [45.8%]; vOPG: 13 [22.0%]; *p* < 0.01).

The comparison of CT, vOPG, and clinical examination is presented in detail in Table 5. The prevalence of TRT was identical between CT and clinical examination (CT: 29.2%; exam: 29.2%; *p* = 1.00). In vOPG, a significantly higher prevalence of TRT was rated compared with the clinical examination (vOPG: 62.5%; exam: 29.2%; *p* ≤ 0.01). The clinical examination revealed a considerably higher prevalence of periodontitis stage 3 or 4 than CT (CT: 41.7%; clinical: 75.0%; *p* = 0.04) or vOPG (vOPG: 37.5%; clinical: 75.0%; *p* = 0.01). Furthermore, clinical examinations showed a high prevalence of increased periodontal probing depth (91.7%) indicating current inflammation and periodontal treatment need. Quite a great proportion had OTN according to clinical examination (91.7%) and vOPG (75.0%; *p* = 0.22) but only a third according to CT (33.3%; *p* < 0.01). 

Regarding periodontitis in the total group, periodontal treatment need was commonly assessed by clinical examination (91.5%). Periodontitis was mainly moderate (grade B: 74.6%) and the mean PISA was 297 ± 264 mm^2^. 

## 4. Discussion

Overall, a high prevalence of clinically and radiographically assessable dental findings requiring intervention (TRT: between 29% and 63%; OTN: between 33% and 92%; pending on examination) was observed in the patient cohort of patients with severe heart disease in the current study. The assessability of vOPG was much more frequently rated as deficient compared with CT and showed a higher prevalence of treatment need. The prevalence of dental findings in CT was much more comparable to clinical examination than vOPG. Imaging underestimated periodontal parameters in general.

The literature on clinical CT imaging (non-dental volume tomography [non-DVT]) for dental pathologies is very limited and this is the first study evaluating the data of pre-existing CTs for dental diagnostic in patients with severe heart failure (HF).

Oral health is an important issue when being evaluated for HTx and LVAD due to their risk for developing systemic infectious complications [5,6,7,8]. Oral bacteria are a potential risk and/or source for systemic infections, especially in the case of (severe and extended) periodontal inflammation [25]. Thus, bacteremia can occur related to dental treatment or daily oral hygiene measures [26,27]. Indeed, oral bacterial DNA was detected in cardiac tissues [28]. The lifelong immunosuppression after HTx leads to a special vulnerability for systemic infections in those individuals [29]. Early dental treatment and the appropriate maintenance of these patients reduces the risks for infectious complications [8]. Additionally, the treatment of patients after HTx or with LVAD also increases the risk for complications during dental therapy [30,31,32,33].

Nevertheless, patients with HF show deficits in oral health. Clinical examinations have shown OTN because of caries for 15% [12] and up to 39% for all causes in [11]. In the present study, the prevalence of OTN differed depending on the detection method: 92% by clinical evaluation, 33% by CT, and 75% by vOPG. Also, TRT differed depending on detection method: 29% by clinical evaluation and by CT and 63% by vOPG. In general, a high prevalence of periodontal treatment need was detected in previous studies, ranging between 69% [11] and 88% [12]. This was evident even for patients with high risk for infections due to LVAD or HTx [12,13,14,15,16,17]. The clinical evaluation of periodontal probing depth identified periodontal treatment need in 92% of the present cohort, which is slightly over this range and therefore in line with the available literature. This shows the necessity for improved interdisciplinary care programs [8,13,34]. The significant discrepancy between different diagnostic methods underscores the necessity of establishing a recommended diagnostic approach. Depending on the diagnostic method used, there can be an over- or underestimation of treatment need, leading to overtreatment or undertreatment. Overtreatment often results in increased tooth loss in this cohort and can delay cardiological treatment, negatively impacting patient care. Conversely, undertreatment poses a higher risk of infectious complications [8] and might lead to later dental treatments with an increased risk of complications [30,31,32,33]. This highlights the need for further research in this area for evaluating appropriate diagnostic strategies. The present study investigated the novel approach of evaluating pre-existing CT imaging as a first dental evaluation. CTs are used for diagnostics for numerous indications including the non-invasive evaluation of a device function in LVAD or the identification of post-operative complications [16]. Accordingly, CT examinations also occasionally cover the orofacial region and exist for 13% of our cohort, which were also initially examined clinically [12,14]. Regarding the technical progress of CT within an ever-shortening acquisition time, its increasing use may be well expected [16]. CT images of the orofacial region are especially interesting since not all dental foci can be identified clinically. Radiographic assessment is necessary for the detection of intraosseous foci such as apical lesions and furcation involvement and can help to identify carious lesions with treatment need [35]. Lots of studies have described the standard use of orthopantomograms in periodontal [36,37] and overall diagnostics [38]. Consequently, radiographs are discussed as a routine part of dental screening before HTx. Nevertheless, there is a lack of clear recommendations. Despite the low radiation exposure caused by dental radiographs [39], radiographic assessments must always respect the principle of ALADA (as low as diagnostically acceptable) being considered [40]. Dental findings, like periapical radiolucency, may frequently be observed incidentally at CT examinations of the head and neck performed for non-tooth-related indications [41]. Thus, the first information on oral health status could already be collected without a dentist visit and without further radiation exposure. For this reason, the existing images should always be evaluated regarding the oral cavity. The use of artificial intelligence (AI) for automated evaluation is a promising approach for dental radiographs [42]. AI could standardize dental evaluations and assist in identifying patients needing further dental assessment, even in cardiological and radiological departments without dental expertise. However, differences in image types and the development of suitable AI models remain significant challenges. Especially, there are few data evaluating the use of AI for 3D images in CT or CBCT [43]. Further research would be necessary to determine AI’s effectiveness with both vOPG and CT images. However, a disadvantage of CT and resulting vOPG images, as also seen in this study, is the presence of metal artefacts. Additionally, CT may be impaired by low resolution and/or low contrast [44]. This study shows the importance of kernel and slice thickness for dental assessability [45,46,47,48,49].

Comparing CT and vOPG, both methods did not differ significantly regarding the assessed severity of periodontitis and apical lesions (Table 4). This is in line with a first proof of concept in eight soft-tissue neck CTs and the corresponding vOPGs [19]. Nevertheless, significant differences between the two methods in the findings regarding the teeth themselves (restoration need and severely damaged teeth), as well as the question of whether the patient has any dental treatment need, were revealed in the present study. For the assessment of the sinuses, however, the superiority of CT may be clearly stated. Orthopantomogram has low efficacy in the diagnosis of sinus disease [50], which is also shown by the clearly higher prevalence of assessed pathologies in maxillary sinuses by CT compared with vOPG in the present study. Furthermore, a significantly higher prevalence of OTN was detected by vOPG. A possible reason for this could lie in the accumulation of layers in the vOPG, which makes small density differences more noticeable, particularly since non-dental CTs that were not optimized for dental hard tissues were used. This could have led to the estimation of a higher number of teeth with caries and consequently the higher OTN in vOPG. Consequently, the stated equal accuracy of both views in the mentioned proof of concept [19] seems not to be valid for all dental pathologies.

Furthermore, especially regarding periodontitis, the clinical evaluation remains essential: Despite radiographic aspects being part of the diagnostic criteria [21], the radiographic bone loss only allows the estimation of past tissue degradation. In contrast, successfully treated stable periodontitis cannot be distinguished from active periodontitis with treatment need with imaging alone [51]. Only a clinical examination by periodontal probing can access the periodontal probing depth and bleeding on probing that differentiates the (in-)stability of periodontitis [51]. Consequently, periodontal treatment need cannot be identified by radiographic evaluation (Table 5). Furthermore, the severity of periodontitis (stage) was underestimated by radiographic assessment, too (Table 2).

On the other side, clinical examination is unable to detect changes located subgingival or within the bone. Therefore, impacted teeth are seldom clinically diagnosable, as they are often positioned subgingival or even entirely within the bone. Similarly, apical lesions are exclusively diagnosed through radiological means. Consequently, adjuvant radiographic imaging is mandatory.

However, the relevance of the different findings differs due to the different systemic clinical impacts and risks for odontogenic complications. Here, a systematic review of dental disease management in cancer patients suggests decisions based on both clinical and radiographic data in combination with reported symptoms [52]: In particular, some findings such as minor caries, asymptomatic third molars, or asymptomatic teeth with periodontal probing depth (<8 mm), mobility (mobility I or II), or with periapical lesions of <5 mm might be considered in dental treatment. Regarding periodontitis, again the clinical examination allows a more detailed evaluation: via PISA, the periodontal inflammatory burden can be estimated [23]. Since PISA correlates with systemic parameters such as c-reactive protein [53] and HbA1c [54], it may enable an estimation of the systemic clinical impact. The mean PISA in the present cohort was below the supposed limit value of 500 mm^2^ that affects systemic inflammatory markers [53].

Altogether, pre-existing CT images should be evaluated regarding dental status and can provide a first overview and information about possible dental foci. They potentially can clarify the question of whether further necessary radiographic assessment may be required. Nevertheless, the clinical examination and its findings should determine if further exams, including dental radiographs, are necessary. Regarding teeth requiring treatment, in this first preliminary study, CTs revealed comparable results to clinical examination. However, the dental evaluation of the CT requires significantly more time than of vOPG [19]. Furthermore, radiologists should be trained specially for dental assessment, and/or logistic solutions must be found for transferring CTs to the dentist. Possibly, vOPG provides a more easily interpretable image for the dentist and could be considered for the initial assessment. Nevertheless, significant differences in the resulting findings must be stated. There are significantly different findings by vOPG compared with both CT and clinical examination. As significantly more vOPGs than CTs were rated as only partly assessable or not assessable, the results of vOPGs must be seen critically. At least for non-dental CTs, the CT itself, instead of the vOPG, should be used for dental assessment. Moreover, the quality in comparison with dental orthopantomograms remains unclear and should be evaluated in further studies. In discussing the different radiographic methods, it is important to emphasize that all statements here refer only to preexisting CT images. While at least conventional dental imaging, such as dental orthopantomograms (OPGs), is necessary for the detection/exclusion of dental foci [35], the use of advanced imaging, such as CT, must be critically evaluated in accordance with the ALADA principle. This principle balances the information added by additional imaging against the potentially harmful higher radiation exposure of CT compared with standard dental radiographs or cone-beam CT (CBCT) [55]. As patients with heart failure are at high risk for systemic infectious complications [5,6,7,8], they may particularly benefit from the early diagnosis of dental foci. Additionally, this patient group often has insufficient access to dental care [13]. Consequently, the decision to use CT may differ from general practice. It would be desirable to assess the long-term impact of dental findings on clinical outcomes. However, demonstrating the effect of dental issues and their treatment on patient outcomes is challenging and would require large cohorts. Given the high risk of systemic infections in heart failure patients, the early detection of dental problems is crucial. Additionally, previous evaluations suggest a potential link between periodontal inflammation and infection-related parameters [56].

Considering all the findings of the current study, especially the different strengths of the distinct diagnostic procedures, dental examination prior to HTx and/or LVAD should include both clinical and radiographic examination. This is in line with another study on patients prior to the implantation of endoprostheses for joint replacement, which also showed the need for clinical and additional radiographic examination to detect potential oral foci of infection [57] and the recommendations of a systematic review of dental disease management in cancer patients [52].

Strengths and limitations: This current study addresses a practically relevant question in the context of the dental care of patients with severe heart diseases. A further strength of the present study is the detailed evaluation of different aspects, the combination of the rating of assessability and the stated treatment need, as well as the evaluation of all aspects by the same calibrated dentist. Nevertheless, several limitations must be announced: Clinically and radiographically assessable findings differ. For example, periodontal treatment need is defined by periodontal probing depth and bleeding on probing [51] that can only be assessed clinically. Furthermore, apical lesions and impacted teeth are radiographic findings, which only in some cases may be suspected by clinical findings. Consequently, the included diagnoses in OTN by clinical and by radiographic assessment differ. Due to the retrospective analysis of patients’ data available from a previous study, no further information on clinical signs for endodontic treatment need could be evaluated. 

In addition, the small number of participants (*n* = 59), especially regarding the smaller subgroup (*n* = 24) for comparison with clinical examination, must be considered for interpretation of the results and limits the generalizability to other cohorts. In addition, some aspects of assessability could only be rated in a small part of the images. This applies especially to the aspects “Sufficiency of restorations” (n_CT_ = 50, n_vOPG_ = 52) and “Quality of root canal treatment” (n_CT_ = 42, n_vOPG_ = 33). The overall power of this study sample is low, indicating that the results must be seen as preliminary; the recruitment or inclusion of more patients was impossible, as the retrospective sample was limited to the number of radiographically examined patients in the study period. Thereby, the initial period of oral examination was between 2017 and 2018, representing a long time span to the retrospective data collection. Furthermore, multiple testing without statistical correction limits the statistical power and must be considered for interpretation. Furthermore, regarding the included patients, it must be noted that mainly male patients (91.5%) were included. This gender imbalance could have influenced the prevalence rates of dental pathologies. However, it does not affect the comparability of the different methods, as the compared methods were applied to the same patients. The use of already existing CTs is part of the scientific question but resulted in very inhomogeneous CTs with partially unfavorable image reconstruction parameters (large slice thickness, soft image kernels). This causes difficulties in assessment and limits transferability to other CTs. Therefore, the present study cannot make any claims about the diagnostic performance of specialized dental CTs. However, it is anticipated that such dental CTs would provide significantly improved assessability and greater diagnostic precision compared with the pre-existing CT scans used in this study. The present study has no gold standard. With the available study design, it is not possible to evaluate the true number of findings. Consequently, it was only possible to show the discrepancies between the different methods and it was not possible to assess the accuracy of the different diagnostic tools directly because it was not possible to assess what were false positive and what were false negative results. Another potential limitation is the long period of (up to three years) between the radiography and oral examinations, as findings can change in this timeline. From the large number of the 454 initially included individuals, only a low number remained for evaluation. Even six months is a long time span between clinical oral examination and CT; however, because the CT images were not performed for dental reasons, this difference between examination and radiology was apparent in this initial study. In a future study setting, a prospective design with a timely CT, ideally on the day of oral examination, appears mandatory. The retrospective character of the study is a methodological limitation. All examinations were performed by the same calibrated dentists under standardized conditions. On the other hand, this must also be seen as limitation the inclusion of multiple (three) evaluators might have strengthened the results and helped to exclude potential bias from evaluator errors. In the present study design, the potential influence of the evaluator cannot be excluded. The evaluator, being a dentist, was more familiar with OPG than with CT images. Nevertheless, we minimized evaluation errors through the calibration process. An inclusion of three calibrated dentists might have strengthened the results but was not considered in the study protocol. For a robust statement, a prospective design with a larger sample size and image acquisition and reconstruction optimized for dental examination with several analyzers would be desirable. For evaluating the additional information of CTs and corresponding vOPGs for dental assessment, a direct comparison to dental orthopantomogram is necessary. Therefore, the findings of this study should only be considered a first preliminary result. Future studies should evaluate the accuracy of the different methods. Therefore, a correct diagnosis must be defined for each tooth. This could be achieved by discussion among multiple experts regarding all methods or by combining in vivo and ex vivo methods. All in all, the present limitations have several implications for the interpretation of the findings. The retrospective study design introduces the potential for selection bias, as the dataset may not be fully representative of the broader patient population. This retrospective approach also limits our control over confounding variables, potentially influencing the outcomes and leading to less accurate conclusions. Furthermore, the heterogeneity of the CTs as well as the small sample size reduces the generalizability of the results and decreases the statistical power of the study. This limitation increases the risk of type II errors, where significant differences between diagnostic methods may remain undetected. Consequently, while our findings provide valuable insights, they should be interpreted with caution. Further research, with larger prospective studies, is necessary to confirm and extend the conclusions, ensuring a more comprehensive understanding of the diagnostic value of CT in detecting dental pathologies.

## 5. Conclusions

Dental findings that require treatment seem to be common among patients with severe heart disease.

None of the available methods are able to indicate reliably true status for all clinically relevant aspects. Nevertheless, already present non-dental CT images that include the craniofacial region performed for other clinical indications may contain valuable information regarding oral health status and should be used for the detection of potential infectious oral foci. However, CT and vOPG seem to underestimate the stage of periodontitis.

The assessability of some vOPGs seems to be insufficient and the resultant findings must be interpreted with caution. The most frequently found reason for impaired assessability were metal artefacts and insufficient resolution, both of which may be improved or even avoided with a CT acquisition optimized for dental imaging.

## Figures and Tables

**Table 1:** Patient characteristics and group comparison.

Variables		Total Included Patients (*n* = 59)	Subgroup with CT ≤ 6 Months from Clinical Exam (*n* = 24)	*p*-Value
Disease [*n* (% of patients)]	HTx	11 (18.6)	3 (12.5)	0.12
	LVAD	35 (59.3)	14 (58.3)	0.55
	HF	13 (22.0)	7 (29.2)	0.09
Age at clinical examination (years) [mean ± SD]		54.9 ± 10.0	52.0 ± 11.8	0.67
Time from HTx to clinical examination (months) [mean ± SD]		28.8 ± 20.2	20.4 ± 30.1	0.11
Time from LVAD to clinical examination (months) [mean ± SD]		29.4 ± 26.9	30.1 ± 29.3	0.24
Sex (male) [mean ± SD]		54 (91.5)	23 (95.8)	0.14
Smoker [mean ± SD]		15 (25.4)	6 (25)	0.78
Time between CT and clinical examination (month) [mean ± SD]		15.0 ± 13.4	3.3 ± 2.3	

Data are mean ± standard deviation or count (percentage). HF: heart failure, HTx: heart transplantation, LVAD: left ventricular assist device, *n*: number of participants, SD: standard deviation.

**Table 2:** Rating of assessability by aspect of computed tomography (CT) and virtual orthopantomogram (vOPG).

Variables [*n* (% of Patients)]	Examination	*n* ^#^	Assessable	Partially Assessable	Not Assessable	Intergroup Comparison (*p*-Value)
Caries	CT	57	5 (8.8)	49 (86.0)	3 (5.3)	**<0.01**
	vOPG	57	8 (14.0)	24 (42.1)	25 (43.9)	
Sufficiency of restorations	CT	50	0 (0.0)	0 (0.0)	50 (100.0)	0.99
	vOPG	52	0 (0.0)	2 (3.8)	50 (96.2)	
Remaining roots	CT	59	27 (45.8)	31 (52.5)	1 (1.7)	0.66
	vOPG	59	26 (44.1)	30 (50.8)	3 (5.1)	
Apical lesions	CT	57	31 (54.4)	26 (45.6)	0 (0.0)	**<0.01**
	vOPG	57	9 (15.8)	42 (73.7)	6 (10.5)	
Presence of root canal treatment	CT	57	41 (71.9)	16 (28.1)	0 (0.0)	**<0.01**
	vOPG	57	8 (14.0)	37 (64.9)	12 (21.1)	
Quality of root canal treatment	CT	24	0 (0.0)	0 (0.0)	24 (100.0)	0.99
	vOPG	33	0 (0.0)	2 (6.1)	31 (93.9)	
Impacted teeth	CT	59	38 (64.4)	21 (35.6)	0 (0.0)	**<0.01**
	vOPG	59	24 (40.7)	34 (57.6)	1 (1.7)	
Maximal horizontal periodontal bone loss	CT	57	30 (52.6)	26 (45.6)	1 (1.8)	**<0.01**
	vOPG	57	12 (21.1)	39 (68.4)	6 (10.5)	
Periodontal vertical bone loss	CT	57	24 (42.1)	30 (52.6)	3 (5.3)	**<0.01**
	vOPG	57	8 (14.0)	24 (42.1)	25 (43.9)	
Maxillary sinus pathology	CT	59	59 (100.0)	0 (0.0)	0 (0.0)	**<0.01**
	vOPG	59	21 (35.6)	33 (55.9)	5 (8.5)	

Data are count (percentage). CT: computed tomography, *n*: number of participants, vOPG: virtual orthopantomogram. Significant values (*p* < 0.05) are highlighted in bold. ^#^ Only those CTs and vOPGs could be included in this rating if the corresponding structures were visible (please see Section 2.2.4).

**Table 3:** Associations between rating of assessability of the images (computed tomography (CT) and virtual orthopantomogram (vOPG)) and the CT reconstruction parameters slice thickness (≤0.75 mm) and kernel hardness (≥45).

Variables	CT			vOPG		
	Slice Thickness	Kernel	Both	Slice Thickness	Kernel	Both
Caries	0.54	**0.02**	**<0.05**	0.08	0.07	**0.04**
Remaining roots	0.17	0.47	**0.03**	0.79	0.20	0.35
Apical lesions	**0.009**	**0.02**	**0.001**	0.32	**0.04**	**<0.01**
Presence of root canal treatment	0.25	0.37	0.21	0.69	**0.006**	**<0.01**
Impacted teeth	0.06	1.00	0.15	0.17	0.65	0.66
Maximal horizontal periodontal bone loss	**<0.001**	0.21	**0.007**	0.80	**0.009**	**0.02**
Periodontal vertical bone loss	**0.002**	0.24	**0.02**	0.09	0.07	**0.04**

Values are *p*-values of Pearson’s chi-squared test describing the association of CT reconstruction parameters and the rated assessability of CT and vOPG. The examined reconstruction parameters were slice thickness (≤0.75 mm) and kernel hardness (≥45). CT: computed tomography, vOPG: virtual orthopantomogram. Significant values (*p* < 0.05) are highlighted in bold.

**Table 4:** Findings according to computed tomography (CT) and virtual orthopantomogram (vOPG).

Variables			CT (*n* = 59)	vOPG (*n* = 59)	*p*-Value
Number of findings per patient [median [0.25 quartile, 0.75 quartile]]					
Restorations needed pp			0 [0, 0]	1 [0, 2]	**<0.01**
Severely damaged teeth pp			0 [0, 0]	0 [0, 0]	**0.04**
Remaining roots pp			0 [0, 0]	0 [0, 0]	0.08
Apical lesions pp	with root treatment		0 [0, 0]	0 [0, 0]	0.63
	without root treatment		0 [0, 1]	0 [0, 1]	0.89
	total		0 [0, 1]	0 [0, 1]	0.77
Partially impacted teeth pp			0 [0, 0]	0 [0, 0]	0.62
Complete impacted teeth pp			0 [0, 0]	0 [0, 0]	0.05
OTN pp			2 [1, 4]	3 [2, 5]	**0.002**
Presence of [*n* (% of patients)]					
OTN			23 (39.0)	47 (79.7)	**<0.01**
Maximal horizontal periodontal bone loss		stage 1	2 (3.4)	7 (11.9)	0.58
		stage 2	28 (47.5)	21 (35.6)	
		stage 3 or 4	26 (44.1)	28 (47.5)	
		Not assessable	3 (5.1)	3 (5.1)	
Periodontal vertical bone loss			6 (10.2)	5 (8.5)	0.99
Maxillary sinus pathology			27 (45.8)	13 (22.0)	**<0.01**

Data are median [interquartile range] or count (percentage). CT: computed tomography, pp: per patient; OTN (presence of teeth with restoration need, severe damage, and/or apical lesions and/or partially impacted teeth and/or remaining roots); vOPG: virtual orthopantomogram. Significant values (*p* < 0.05) are marked in bold.

**Table 5:** Treatment need as identified by computed tomography (CT), virtual orthopantomogram (vOPG), and clinical dental examination.

Variables		CT (*n* = 24) ^#^	vOPG (*n* = 24) ^#^	Clinic (*n* = 24) ^#^	*p*-Value		
					CT vs. vOPG	CT vs. Clinic	vOPG vs. Clinic
Presence of TRT [*n* (% of patients)]		7 (29.2)	15 (62.5)	7 (29.2)	**<0.01**	1.00	**<0.01**
Number of TRT per patient [median [0.25 quartile, 0.75 quartile]]		0 [0, 1]	1 [0, 2]	0 [0, 1]	0.05	1.00	0.05
Periodontitis stage [*n* (% of patients)]	1	2 (8.3)	4 (16.7)	1 (4.2)	0.38	**0.04**	**0.01**
	2	11 (48.5)	9 (37.5)	5 (20.8)			
	3 or 4	10 (41.7)	9 (37.5)	18 (75.0)			
	Not assessable	1 (4.2)	2 (8.3)	0			
Presence of OTN [*n* (% of patients)]		8 (33.3)	18 (75.0)	22 (91.7)	**<0.01**	**<0.01**	0.22

Data are median [interquartile range] or count (percentage). Clinic: clinical dental examination; CT: computed tomography; OTN: overall treatment need (CT/vOPG: presence/number of teeth with TRT or apical lesions or partially impaction; clinic: presence of TRT or periodontal treatment need); TRT: teeth requiring treatment due to caries, insufficient restorations, severe damage or remaining roots; vOPG: virtual orthopantomogram. Significant values (*p* < 0.05) are highlighted in bold. ^#^ Only those patients with CT within 6 months of clinical examination were considered for this comparison.

## Data Availability

The datasets used and/or analyzed during the current study are available from the corresponding author on reasonable request. The data are not publicly available, because of the pseudonymization and data protection guidelines according to the ethics approval.

## Abbreviations

Clinic	clinical dental examination
CT	computed tomography
HF	heart failure
HTx	heart transplantation
LVAD	left ventricular assist device
OTN	overall treatment need (CT/vOPG: presence/number of teeth with TRT, apical lesion or partially impaction; clinic: presence of TRT or periodontal treatment need)
TRT	teeth requiring treatment (presence/number of teeth with restoration need due to caries, insufficient restorations, severe damage, or remaining roots)
vOPG	virtual orthopantomogram
